# The Clinical Anatomy of Endometriosis: A Review

**DOI:** 10.7759/cureus.3361

**Published:** 2018-09-25

**Authors:** Yusuf Alimi, Joe Iwanaga, Marios Loukas, R. Shane Tubbs

**Affiliations:** 1 Anatomy, St. George's University School of Medicine, St. George's, GRD; 2 Medical Education and Simulation, Seattle Science Foundation, Seattle, USA; 3 Anatomy, St. George's University, St. George's, GRD; 4 Neurosurgery, Seattle Science Foundation, Seattle, USA

**Keywords:** endometriosis, pelvic pain, dysmenorrhea, diagnosis, management

## Abstract

Endometriosis is a gynecological disorder identified by the presence of ectopic endometrial tissue outside the uterus. Largely, it affects reproductive-aged women and is a major cause of infertility. Clinical manifestations of endometriosis include dyspareunia, cyclic menstrual pain, chronic pelvic pain, and dyschezia, all of which can affect the patient’s quality of life and health severely; therefore, it is paramount that medical treatment is initiated as soon as endometriosis is suspected clinically. In this review, we examine the known anatomic principles of endometriosis in the literature and outline ways to manage patients with this condition better.

## Introduction and background

**Figure 1:**
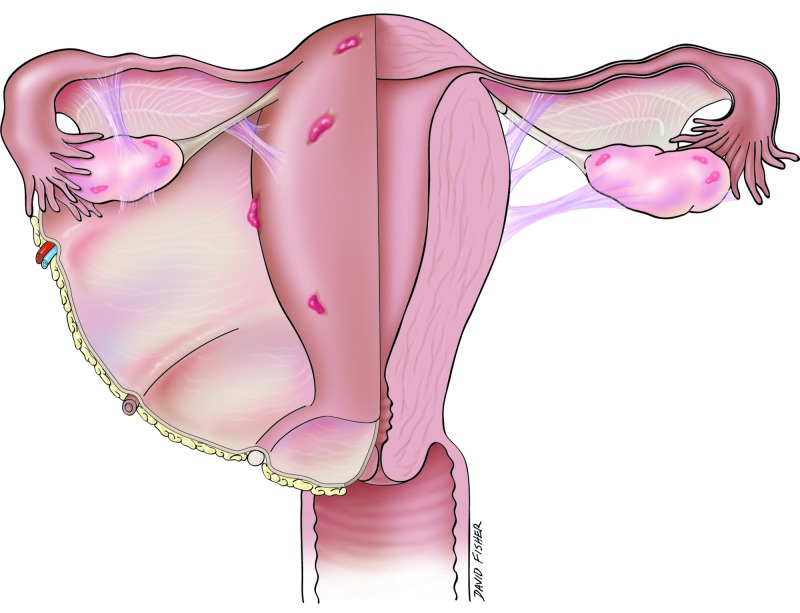
Schematic drawing of areas often involved in endometriosis.

Endometriosis is defined as the presence of endometrium in an abnormal or ectopic location (Figure 1). Histologically, it is the presence of endometrial-like tissue or glands outside the uterine cavity [1-5]. It is a gynecological disorder dependent on hormones observed most commonly in reproductively active women [2,4-5]. The ectopic endometrial tissue responds to hormonal stimulation and undergoes cyclic growth and shedding. Without a way to drain, this causes internal accumulation of blood. Endometriosis is associated often with dyspareunia, cyclic menstrual pain, and pelvic pain [4]. These painful episodes can have a negative effect on the quality of life of patients with this condition experience.

Endometriosis often can present without symptoms, and therefore, is missed frequently as a diagnosis. Its prevalence is between 5% and 10% in premenopausal women and can reach as high as 35% in women suffering from subfertility, as it can be a major cause of infecundity [2,3,6]. The known risk factors for endometriosis include menarche beginning at less than 11 years of age, as well as heavy and prolonged menses [2,6-7]. These two factors increase the extrauterine environment’s exposure to menstrual blood and endometriosis risk. The most common sites of pelvic endometriosis are the ovaries, uterine ligaments (largely broad and uterosacral ligaments), pouch of Douglas, and fallopian tubes [2-4]. Endometriotic implants also have been found in extra-pelvic locations, including the gastrointestinal tract, lungs, diaphragm, abdomen, and pericardium [2-4, 8].

Three major forms of endometriosis are found in the pelvic region: ovarian, peritoneal, and infiltrating endometriotic lesions [2,9-10]. Morphologically, there are three types of endometriotic lesions: white, red, and black lesions. The red lesions represent activity with a high level of vascularization, while the whitish lesions are later phases of red lesions that have undergone a process of inflammation and fibrosis. The classical black lesions are attributable to cyclic tissue decomposition and healing with the subsequent formation of scar tissue [2,4,6].

## Review

Classification and staging

The revised American Fertility Society score (high-resolution), now referred to as the American Society for Reproductive Medicine (ASRM) criteria, for classifying the severity of endometriosis is used most widely among clinicians [6,11-13]. The ASRM is based upon the recordings of operating room findings and comparisons of the efficacy of therapeutic interventions [6]. The ASRM criteria classify endometriosis on a point score from Stage I (minimal) to Stage IV (severe) based on the location and size of the lesions seen during the surgical procedures (Table 1) [12].

**Table 1:** The staging system considers the morphological appearance, number, location, and size of the endometriotic lesion and assigns scores according to these factors. The higher the score, the greater the severity of endometriosis.

THE REVISED AMERICAN SOCIETY FOR REPRODUCTIVE MEDICINE CRITERIA FOR ENDOMETRIOSIS STAGING	
STAGE I (MINIMAL)	POINT SCORE BETWEEN 1-5
STAGE II (MILD)	POINT SCORE BETWEEN 6-15
STAGE III (MODERATE)	POINT SCORE BETWEEN 16-40
STAGE IV (SEVERE)	POINT SCORE BETWEEN > 40

One of the major drawbacks of the ASRM classification system is its inability to predict the probability of conception after surgery, which is of significance for patients trying to conceive. This has led to the development of newer classification systems, such as the Endometriosis Fertility Index (EFI) [11,14]. The EFI combines the point scoring system of the ASRM with fertility information post-surgery. Patients are assigned a score ranging from zero to 10 and after three years; those with scores between 0 and 3 had a 10% probability of conception, while those with scores between 9–10 had a 75% chance of conceiving. The EFI does not consider in vitro fertilization (IVF) treatments after surgery and has been used widely in various studies as a predictor of pregnancy rate and live births following laparoscopy [11,15-17].

Pathophysiology 

Myriad hypotheses and theories have been proposed for the pathogenesis of endometriosis. These include the implantation or metastatic, metaplasia, induction, and endometriosis disease theories, and most recently, the stem cell theory [2,4,9,18]. The most widely accepted theory is implantation, which involves the establishment of an early lesion in the uterus that serves as a nidus for endometrial tissue proliferation [2,18-20]. The endometrial tissue then spreads to other pelvic regions via retrograde menstruation, with subsequent attachment to, and invasion of, the peritoneum [2,5,18-20], which leads inevitably to the establishment of ectopic endometrial tissues outside the uterus. However, because most women experience retrograde menstrual flow, while only approximately 10% suffer from endometriosis, there is more to endometriosis than retrograde menstrual flow, which is what led to the proposal of the stem cell theory [2,18-20]. As endometrial progenitor cells have been found to be shed during the menstrual cycle, the retrograde menstruation theory was expanded, and it was established that these stem cells spread to the peritoneum via this process [18-20]. The cell type that initiated the spreading process explains the different grades of endometriosis [18]. Endometriosis that begins with endometrial stem cells tends to be more severe than that which originates from the spread of more differentiated cells. This is attributable to the stem cells’ plasticity and the retained ability to attach, differentiate, and proliferate extensively, and therefore exhibit a greater tendency to colonize an ectopic site [2,18-20]. Women with heavier and longer menstrual flow or abnormal uterine bleeding also have been proposed to be more susceptible to endometriosis, as these conditions increase the extra-uterine environment’s exposure to endometrial tissue [2,5].

Anatomy and pain-related endometriosis

The uterus develops embryologically from the paramesonephric ducts (also referred to as the Mullerian ducts), which in the absence of the Mullerian inhibiting factor, present typically in males, proliferate and differentiate and give rise to the fallopian tubes, uterus, and upper third of the vaginal canal [21]. In endometriosis, the endometrial tissues spread from the uterus to other pelvic organs, such as the ovaries and fallopian tubes [1-2]. Deep infiltrating endometriosis (DIE), defined as the presence of endometriotic implants greater than 5 mm beneath the peritoneum, is a major indicator of the severity of pain women with this disorder experience [22-23]. A retrospective observational study of women who underwent a surgical procedure to remove DIE lesions showed that most women with this condition present with multiple forms of pelvic pain, in which the bowel is the most frequent location of pain, followed by the uterosacral ligaments [22]. Avila et al. observed further that deep dyspareunia was associated largely with vaginal and rectovaginal septum DIE, with dyschezia related closely to adhesions in the cul-de-sac [22]. DIE also has been reported in the urinary tract, with bladder and/or ureteral involvement, and is prevalent in 6% of women with pelvic endometriosis [23].

Pain mechanism

Two types of pain—visceral and somatic—are the primary symptoms endometriosis patients experience and can be quite complex [4,24]. Visceral pain arises from the inner organs, such as the bladder, uterus, and the rectum, while somatic pain is experienced when sensory nerves located in the skin and deep tissues are triggered [24]. Endometriosis pain is a complicated combination of both types of pain all patients experience to different degrees [24], which contributes to the complexity of treatment seen in this disorder.

The debate whether the pain mechanism endometriosis patients experience has a neuropathic or nociceptive origin continues, but more evidence supports the latter [24]. Typically, an injury to or disease of the somatosensory nervous system causes neuropathic pain, which can be differentiated from non-neuropathic causes by the absence of an inciting nociceptive stimulus [25]. In contrast, actual or imminent tissue damage and the subsequent stimulation of nociceptive neurons causes nociceptive pain [24-26].

The argument for neuropathic pain is weakened by the disappearance of painful symptoms upon surgical removal of the endometriotic lesions, and the central sensitization patients with endometriosis perceive can be attributed to the inflammatory processes that occur after the non-neural tissue damage in nociceptive pain [24]. However, patients can experience neuropathic pain when they have undergone a surgical procedure because of the nerve injury that may occur during this process. This is suggested to be a cause of the recurrent pain in endometriosis patients whose lesions have been removed [24].

The perineural spread theory

Roth, who proposed the perineural spread of endometriosis into the inferior hypogastric plexus, developed the theory of the spread of endometriosis to nerve tissues in the pelvis [27]. Since its inception, other studies have demonstrated the involvement of nerves originating from the lumbosacral plexus, including the obturator and sciatic nerves [1,28-30]. De Sousa et al. demonstrated the spread of endometriosis from the uterine cavity along the autonomic nerves in the pelvis into the lumbosacral plexus [28]. Further spread of the endometriotic lesions into the spinal nerves and even the dura of the spinal cord was proposed to be a possible etiology of DIE [1,28]. The perineural spread theory is not limited to the pelvic nerves, as the involvement of the central nervous system also has been reported [28]. This includes the cerebellar vermis of the brain, frontal and parietal lobes, cauda equina, and conus medullaris [31-35]. The supporting evidence for endometriosis spread via the perineural approach is endometriotic lesions’ expression of nerve growth factor and the presence of the nerve growth factor (NGF) receptor (Trk-A) on the pelvic nerves [36]. Anaf et al. proposed that the expression of NGF in endometriosis and Trk-A on neural tissues results in the proliferation of the nerves that causes increased nerve sensitization and pain [36].

Clinical manifestations, diagnosis, and imaging in endometriosis

Endometriosis can be asymptomatic, but the most common clinical manifestations include cyclic menstrual pain, chronic pelvic pain, dyspareunia, menorrhagia, and dyschezia [6,37]. The pelvic pain in women with endometriosis is described as pain before the onset of menses and deep dyspareunia that worsens upon menstrual flow. Sacral and lower backaches during menses also can be present [6]. The first diagnostic evaluations in these patients are physical examinations and pelvic ultrasound [17,38]. Physical examination findings might include uterosacral ligament tenderness and nodularity, as well as the presence of an adnexal mass that most likely is an ovarian endometrioma [6,38]. Ovarian endometriomas, also known as chocolate cysts, are large, fluid-filled cysts that develop on the ovary because of endometrial tissue deposition via retrograde menstruation [6].

A presumptive clinical diagnosis of endometriosis can be made based on the clinical manifestations described above. However, the gold standard for diagnosing endometriosis is laparoscopy with biopsy to demonstrate the histological presence of the endometrial tissues [6,37]. Chocolate cysts can be visualized via ultrasonography, and DIE can be detected using transvaginal ultrasound [6,39]. Pelvic magnetic resonance imaging (MRI) has been touted as a better imaging modality in patients with deep infiltrating pelvic endometriosis, as it offers high-resolution images with excellent tissue characterization [9,17]. MRI also allows endometriotic lesions and implants to be visualized, which might not be visible via ultrasonography and permits a complete scan of all the pelvic compartments [9]. This is highly important, especially in individuals who are about to undergo a surgical procedure to remove the endometriotic implants, as it decreases the likelihood of missing a lesion or implant, and therefore, leads to a better surgical outcome.

Infertility in endometriosis

Infertility is a common complication that occurs in women with moderate to severe endometriosis [3,11,40]. Prescott et al.’s large cohort study showed that women less than 35 years of age with endometriosis have an increased risk of infertility [7], which can be secondary to endometriotic lesions or implants’ distortion of the normal pelvic anatomy [11,40]. Women with endometriosis also have been shown to have an increase in macrophages and specific cytokines in the peritoneal fluid [11,40]. This is attributable to the acute inflammation the presence of ectopic endometrial implants induces. These macrophages maintain a state of chronic inflammation and the formation of adhesions, as well as angiogenesis [11]. The increased macrophages and scarring have been postulated to interfere with normal sperm motility and ciliary function of the fallopian tubes [11,40]. The development of adhesions also might obstruct the normal tubal transport, thereby causing infertility [11,40]. In the absence of any of the above, other mechanisms that have been proposed as a cause of decreased fertility in women with endometriosis include perturbed folliculogenesis secondary to pituitary dysfunction, luteal phase defects, progesterone resistance, and anti-endometrial antibodies [11].

Unusual cases of endometriosis

Endometriosis has been known to spread to extra-pelvic sites, including the gastrointestinal tract, lungs, liver, pericardium, and even the brain [2,8,35]. A case report by Fluegen et al. also demonstrated ectopic endometrial tissues in the liver [41]. The patient was a 32-year-old woman who presented with non-cyclical upper right quadrant pain in the abdomen with an initial diagnosis of lobulated intrahepatic cysts. She underwent laparoscopic procedures to remove the putative cyst; however, symptoms persisted, which prompted further evaluations [41]. The diagnosis of intrahepatic endometriosis was confirmed via histological analysis and immunostaining after laparoscopic pericystectomy [41]. This is among the few unusual cases of extra-pelvic endometriosis, in which the patient did not present with the typical “cyclic” pain that might have aided the diagnosis. Intrahepatic endometriosis also has been found in post-menopausal women, suggesting that this condition is not limited to women of reproductive ages [41]. Cases of thoracic endometriosis presenting as hemoptysis and spontaneous pneumothorax also have been reported in the literature [42]. These few examples indicate the complexity of endometriosis and the way atypical cases of this disorder might present. They also serve as a caveat to clinicians to be aware of rare atypical endometriosis presentation and indicate this as a probable differential diagnosis, especially in female patients with recurrent pain in extra-pelvic locations devoid of a certain etiology.

Management and treatment

The goal of management in patients with endometriosis is early diagnosis and focused treatment to prevent disease progression and improve patient's quality of life [37]. Once a presumptive diagnosis of endometriosis has been made, medical therapy should be initiated.

Medical Therapy

It is worth noting that medical therapy in patients with endometriosis is non-curative and serves only to suppress disease progression [6-7,37]. Mild pain can be managed via the use of nonsteroidal anti-inflammatory drugs (NSAIDs), oral contraceptives, and progestins [6-7,43]. The initial hormonal therapy for pain secondary to endometriosis can be either a combined hormonal contraceptive or the levonorgestrel-releasing intrauterine system [17]. The second line of hormonal therapies is low-dose progestin, which antagonizes estrogen’s hormonal effect in the endometrial tissues, as well as gonadotropin-related hormone antagonists (GnRHas) that modulate the signaling of the hypothalamic-pituitary axis, thereby decreasing estrogen release [6,17]. As mentioned earlier, infertility is a major complication of endometriosis and cannot be treated via medical therapy, as all medical treatments available for endometriosis work by suppressing ovulation [7,37].

Surgical Therapy

Surgical management is the primary treatment for infertile patients with endometriosis, as it can improve the patient’s probability of spontaneous conception or pave the path for in vitro fertilization in patients with severe endometriosis [6,37]. Patients with severe pain refractory to medical therapy also can benefit from surgery, as shown by the pain relief experienced by up to 95% of patients who underwent laparoscopy to excise lesions [6,37]. Conservative surgery, including laparoscopy for definitive diagnosis, lysis of adhesions, and removal of visible implants, is the primary approach to symptomatic endometriomas, but special attention must be given to these patients because of the high risk of injury to the ovaries and compromise of the ovarian reserve, as well as the potential hindrance to future fertility [6,37,44]. Other surgical techniques, such as laparoscopic uterine nerve ablation that disrupts the efferent nerve fibers present in the uterosacral ligaments and presacral neurectomy that disrupts sympathetic innervation to the uterus at the level of the superior hypogastric plexus, have been performed with varying degrees of success [6,44]. Hysterectomy also has been suggested for women with severe, debilitating, and refractory endometriosis who do not wish to become pregnant and in whom other therapeutic measures have failed [6,44]. Postoperative suppressive medical therapy is advised in patients who have undergone surgical procedures for endometriosis, as it offers longer pain relief compared to surgery alone [6,14,44-45]. This includes the use of combined hormonal contraceptives or the 52-mg levonorgestrel-releasing intrauterine system [17]. After surgery, patients can be evaluated with the endometriosis fertility index to determine their probability of future conception [11,17].

It is noteworthy that although surgery might increase the patient’s likelihood to conceive, Prescott et al. concluded that it provides only approximately an 8% increase in the conception rate among patients with Stages I-II endometriosis [7]. Although evidence of the reproductive benefits of surgery in endometriosis patients with advanced staging is lacking, a retrospective study has shown that women with moderate to severe endometriosis who underwent surgical resection and evaluated using EFI post-surgery can have as much as a 91% live birth rate after five years [16].

Endometriotic lesions that recur after surgery have been found to occur in the same vicinity as the previous lesions and patients who undergo conservative surgery have a higher likelihood of recurrence, as some small residual implants might remain post-operatively [45]. Koga et al. proposed that the prolonged use of suppressive medical therapy post-surgery (greater than 6 months) can prevent recurrence of dysmenorrhea in most patients, but has little to no effect in controlling recurrent chronic pelvic pain or dyspareunia [45]. They attributed medical suppressive therapy’s efficacy in dysmenorrhea to the fact that dysmenorrhea results from endometrial bleeding, and the therapy available works by suppressing endometrial proliferation. However, this is not the case with chronic pelvic pain and dyspareunia, as multiple physiopathological factors play roles in the development of these latter conditions [45].

## Conclusions

Endometriosis is a complex disorder characterized by pain and infertility that, if not treated properly, can compromise the patient’s quality of life and health significantly. Clinical suspicion of endometriosis should be met with the appropriate medical therapy and patients informed about complications that might arise from this condition, primarily infertility. Further, knowledge of the different atypical presentations and imaging modalities used to diagnose endometriosis is the clinician’s responsibility, and the significance of its awareness cannot be understated.
